# Multi-database study of multiple sclerosis: identification, validation and description of MS patients in two countries

**DOI:** 10.1007/s00415-019-09238-8

**Published:** 2019-02-18

**Authors:** Rebecca Persson, Sally Lee, Marianne Ulcickas Yood, Michael Wagner, Neil Minton, Steve Niemcryk, Anders Lindholm, Amber Evans, Susan Jick

**Affiliations:** 1 Boston Collaborative Drug Surveillance Program, Lexington, MA USA; 2 Boston University School of Public Health, Boston, MA USA; 3 Celgene Corporation, Summit, NJ USA; 4 Celgene Europe Limited, Uxbridge, UK; 5 Health ResearchTx, LLC, Trevose, PA USA; 6 Department of Neurology, Naval Medical Center Portsmouth, Portsmouth, VA USA; 7 EpiSource, LLC, Newton, MA USA

**Keywords:** Multiple sclerosis, Validation, Prevalence, Comorbidities

## Abstract

**Objective:**

To describe the resources and methods used to identify and validate multiple sclerosis (MS) and match non-MS patients in each of the two databases, and to characterize their demographics, comorbidities and concomitant medications.

**Methods:**

This study was conducted in two separate electronic medical databases, the United States Department of Defense (DOD) military health care system and the United Kingdom’s Clinical Practice Research Datalink (CPRD) GOLD. We identified patients with a first recorded diagnosis of MS in 2001–2016 (CPRD) or 2004–2017 (DOD) and matched non-MS patients using algorithms appropriate to each database. We describe patient symptoms, comorbidities, and medication use at the time of the MS diagnosis and compared them to the non-MS cohort.

**Results:**

We identified 8695 patients with MS and 86,934 matched non-MS patients in the DOD database and 6932 patients with MS and 68,526 matched non-MS patients in CPRD GOLD. Most MS patients were female (around 70%) and were diagnosed before age 60 (88%). MS patients had higher prevalence of depression and other psychiatric conditions at MS diagnosis compared to non-MS patients. Epilepsy, fractures and infections were also more common. MS patients had many expected symptoms and treatments documented in their records prior to the MS diagnosis.

**Conclusion:**

These results are consistent between the two databases, as well as with previous studies of MS. Future analyses of these patients’ experience after MS diagnosis will provide valuable insights into disease and treatment patterns in relation to risk of chronic diseases and mortality.

## Introduction

Multiple sclerosis (MS) is the most prevalent permanently disabling neurological disease among young adults in Europe and North America, and is associated with diminished quality of life and high socio-economic cost [1–3]. There have been recent important advancements in the treatment of this condition, but the long-term implications of these treatments on comorbidities and mortality are not well understood. We conducted a study to follow MS patients and matched non-MS patients to assess changes in comorbidities and drug use in the two cohorts during follow-up and over the progression of the disease in the MS patients. The study covered the period 2001–2017 and was conducted separately in three databases across three countries to further compare patient characteristics, treatments, and outcomes in different patient populations. In this paper, we describe the Department of Defense military health system database (DOD) (United States (US)), and the Clinical Practice Research Datalink (CPRD) GOLD (United Kingdom (UK)). Information on the third contributor to this study (Swedish registry data) will be reported at a later date. The objective of this manuscript is to describe the resources and methods used to identify and validate the MS patients and the matched non-MS cohorts in each of the two databases, and to characterize the demographics of MS patients in the US and the UK, as well as comorbidities and concomitant medications at first MS diagnosis, and to compare these with those of a matched general population.

## Methods

This study was conducted in two separate databases to describe patients with MS and their variations in comorbidities and concomitant medications at the time of MS diagnosis across different countries. Both data resources are described below. See Table 1 for a comparison of database details and coding systems used in each.

**Table 1:** Characteristics of the two study databases

	United States—DOD	United Kingdom—CPRD GOLD
Database size	~ 10 million patients with up to 14 years of follow-up	~ 10 million patients with up to 30 years of follow-up
Number of MS/matched non-MS patients	8695/86,934	6932/68,526
Data type and source	DOD claims (100%) and EMR data (40%)	General practitioner EMR data (100%) with links to hospital data (60%)
Function of diagnosis codes within database	Reimbursement (includes screening, rule-out and “true” diagnoses)	Medical record (confirmed diagnoses)
Study period	2004–2016	2001–2016
Codes used for diagnosis	ICD-9, ICD-10	Read codes for GP data and ICD-10 for hospital data
Codes for procedures	CPT	Read codes or OPCS in hospital data
Codes used for medications	NDC, HCPCS	Gemscript, OPCS
MS treatment	Complete through NDC, HCPCS, and CPT	Limited to treatments provided by GP or in-hospital infusions
Access to original records	Through DOD physician	Through questionnaire responses

### Database descriptions

#### Department of Defense (US)

The US study was conducted utilizing clinical and administrative data from the DOD military health care system and Health ResearchTx, a health research organization that works with the DOD to provide healthcare information for research purposes [4]. The DOD data are a US-based, ongoing longitudinal database with health information on approximately 10 million active beneficiaries (49% female). It is comprised of data contributed by members of the US DOD, retirees and dependents. Only 14% are active service members. Data are available and of sufficient quality for use in research from October 2003 to 2017 and the average follow-up per patient is 8 years.

For patients who receive medical care at DOD facilities, the data contain virtually complete electronic medical records (EMR), including demographic information, prescription details, clinical events, referrals, hospital admissions, health care resource use, laboratory results, and vital patient characteristics, such as blood pressure, smoking, alcohol consumption and height and weight, and have well-documented validity. The data for all patients in the database are recorded in enrollment, pharmacy, and inpatient/outpatient (diagnoses, procedures) files. Drug prescriptions are coded using the National Drug Code (NDC) and each drug claim includes information on the specific product dispensed, the date, quantity, length of supply and refills provided. All medical events and procedures are coded with the International Classification of Diseases (ICD)-9/10 coding system, DRG codes, and CPT codes (procedures). These include inpatient, outpatient and emergency encounters. Up to 20 diagnoses are listed under each hospitalization in a DOD hospital, and 12 in private sector/civilian hospitals. Around 40% of all patients receive medical care at DOD facilities while the remaining 60% are seen for care in the civilian or private sector. Deaths are identified for all patients through a master death file that is updated by a recurring Social Security Death Index (SSDI) feed from the Social Security Administration.

There is also access to chart review of the original records to validate outcomes and obtain additional clinical details. The long average follow-up time and relatively large and stable population are important features of the data. Finally, the DOD covers patients in all 50 states and is thus geographically representative of employed US citizens and their families.

### Clinical Practice Research Datalink (CPRD) GOLD

CPRD GOLD, established in 1987, is a large, prospectively collected, anonymized medical record database encompassing over 500 UK general practices, covering over 10 million patients and over 65 million person-years of follow-up. It is a population-based resource broadly representative of the UK population (including England, Scotland, Wales, and Northern Ireland) in terms of age, sex, and minority distribution [5]. CPRD GOLD contains virtually complete electronic medical records, including demographic information, prescription details, clinical events, referrals, hospital admissions, laboratory results, health care resource use, and lifestyle details, such as smoking, alcohol consumption and height and weight, and has well-documented validity. CPRD GOLD data are recorded in multiple files, including the registration, drug, events (diagnoses, procedures), laboratory, and patient characteristics files. The Drug file contains detailed information on all drugs prescribed by the general practitioner using the Gemscript coding system. Drug details include the date, precise drug formulation, strength, and quantity of drug prescribed, and the dosing instructions. In certain instances, a specialist may prescribe a course of treatment; these prescriptions are not captured in the database, though future drug therapy is usually directed through the general practitioner and thus captured in the database. Treatments that do not result in a written prescription, such as infusions, are also only rarely captured. The Event file contains all clinically relevant patient diagnoses (inpatient, outpatient, and emergency department) along with the date of the event. In addition to usual care in the office, general practitioners are required to enter the indication for any new drug therapy, as noted above, as well as all diagnoses resulting from hospitalizations, consultations, or emergency medical care. Because the general practitioner is the primary caregiver for all patients in the National Health Service (NHS), all consultants are required to send a letter to the general practitioner describing the relevant clinical events and final diagnoses whenever a patient is seen in hospital or by an outpatient specialist. The key contents of these letters, primarily clinical diagnoses, are then entered into the computer file by the general practitioner. CPRD GOLD uses the Read coding system which provides more detailed clinical codes than ICD. There is also a file with Additional Clinical Details which contains patient information such as blood pressure, height, weight, smoking status, alcohol use, and other lifestyle characteristics. Information on laboratory tests and results is available in a lab file. Finally, additional clinical information and data validation can be obtained through questionnaires to the GPs. These data are not claims based.

In addition to the information in the CPRD GOLD record, it is possible to link around 60% of all CPRD GOLD patients to hospitalization and death registry data (for practices from England only, not Wales, Scotland or Northern Ireland). The Hospital Episode Statistics (HES) contain details of admissions to NHS hospitals in England including dates of admission and discharge, the primary diagnosis, additional diagnoses, and procedures. These data include treatments, such as infusions, provided in outpatient hospital clinics. Diagnoses are recorded using ICD-10. The death registry contains details of all deaths in England through mid-2017 including primary and secondary causes of death as noted on the patient’s death certificate. We also received the ONS death registry data for all patients in the study population with registry linkage. These data include the primary cause of death and up to 15 secondary causes, coded with the ICD 10 coding system.

The UK provides a unique medical environment for epidemiological research because of the universal health system which minimizes selection bias related to differential access to healthcare, and because all patient care is centralized with the general practitioner. Information from all inpatient and outpatient medical encounters is reported to the general practitioner in the form of consultant and discharge letters, and is coded in the electronic patient record resulting in virtually complete ascertainment of all medical outcomes for all patients in CPRD GOLD. Thus, concern about missing events treated by specialists is minimized in this study. The results of validation studies found the data to be of high quality and completeness [6, 7]. Access to original records via questionnaires and death registry data, long average follow-up time (> 8 years) and a relatively stable population enrolled in a state-controlled health system are all important features of the data. All patients in this study were identified from the general practice setting and, therefore, are representative of patients attending general practice care in the UK. We have used the CPRD GOLD data for many studies, including many prior studies of MS [8–15].

### Study population

Within each database, we identified all people with a first recorded diagnosis of MS in years 2001–2016 (CPRD) or 2004–2017 (DOD). To identify newly diagnosed cases of MS, we required at least 1 year of enrollment in the database before the first MS diagnosis. Cases were then validated through multiple steps that varied according to database. The objective of data recording is fundamentally different in the CPRD compared to the DOD database. One is a GP electronic record (CPRD) while the other is a claims database (DOD), thus the recording of diagnoses differs greatly between the two and the criteria for identifying MS patients, and defining comorbidities also differs (Table 1). These differences are reflected below in the selection and validation of study outcomes.

In the DOD, we included all patients with at least one diagnosis of MS or demyelinating disease and at least one prescription for a MS disease-modifying treatment or dalfampridine. We then classified these patients as probable or possible MS patients. Probable cases were those that had at least ten diagnoses of MS or demyelinating disease on different dates and at least five prescriptions for a MS disease-modifying treatment or five or more MS diagnoses on different dates and at least ten disease-modifying treatment prescriptions or infusion codes. Possible cases were those with demyelinating disease codes (without any MS codes) plus disease-modifying treatment prescriptions, or one to ten MS diagnosis codes and one to ten disease-modifying treatment prescriptions. In US claims databases, a code for the presumptive diagnosis is included each time a test is conducted or a visit occurs to further investigate the disease. Thus, in the DOD database, patients with fewer than ten codes for MS were less likely to have true MS. We reviewed the electronic records from a sample of all MS patients in collaboration with a DOD physician, to validate the MS case selection process.

CPRD GOLD is an EMR and not a claims database; consequently codes are entered for purposes of the GP’s medical record keeping: GPs record confirmed not provisional diagnoses unlike in the DOD database. Once the diagnosis has been entered in the patient record it is not necessary to repeat it and 66% of MS cases in the CPRD had only one or two codes for MS. Furthermore, MS disease-modifying treatments are not regularly captured in the CPRD because many of them are administered as infusions outside the GP’s surgery. Thus, to have confidence that a patient truly had MS we validated MS cases in the CPRD through supporting codes. Probable MS cases were those whose records contained two or more MS diagnosis codes on different dates plus treatment and/or symptom codes. Possible MS cases were those whose records contained (1) at least one MS diagnosis plus codes for symptomatic treatments or symptoms that may or may not have been related to MS, or (2) the record contained two or more MS diagnosis codes on different dates with no supporting treatment or symptom codes. Unlikely cases of MS included those whose records contained one MS diagnosis code only and no supporting treatment or symptom codes, or the record contained an alternate diagnosis such as prior stroke.

In light of the small number of MS diagnoses included and the absence of treatment information in GP records, we conducted a validation study in the CPRD population to assess the MS case algorithm. We requested and received questionnaires on a sample of patients in the CPRD to validate our case algorithm. Thus, despite the differences in the databases and the basis for recording diagnoses in each, we were able to develop and validate database-appropriate case selection procedures customized to each resource.

To exclude uncertain or incorrect MS diagnoses, cases in the CPRD and DOD were excluded where (1) amyotrophic lateral sclerosis was coded at any time in the record or (2) where another alternate diagnosis was present at some time in the record and only one MS code (CPRD) or less than five MS codes (DOD) were present, or (3) where only one MS code (CPRD) or less than five MS codes (DOD) were present and the patient had a code for stroke or transient ischemic attack at any time prior to or up to 6 months after the MS code.

The MS diagnosis date (cohort entry date) was the date of the first recorded MS code in both the DOD and CPRD GOLD databases. All patients with MS were then matched to up to ten patients without MS on age, sex, month and year of cohort entry in the database, and geography (same practice in the CPRD; same region in the DOD).

### Study outcomes

In each database, for each MS and non-MS patient, we identified MS symptoms, comorbidities and concomitant medications present at the time of cohort entry (MS diagnosis date) or at the matched date (month and year) for the non-MS patients. See Tables 3, 4, 5 and 6 for outcomes included.

A patient was considered to have a chronic study comorbidity, such as asthma, epilepsy or liver disease, if the disease was recorded at least once (CPRD) or at least five times (DOD) any time prior to or on the cohort entry date. Chronic study outcomes in the DOD require more recordings because of the claims nature of the data and to avoid misclassification of outcomes. Treated depression, hypertension and type II diabetes were defined as the presence of at least one diagnosis code and one respective prescription within 90 days of each other. In the CPRD, cancer was defined as one or more diagnosis codes for cancer or history of cancer. In the DOD, cancer was defined as five or more diagnosis codes within 6 months of each other, or ten or more diagnosis codes at any time before cohort entry, or at least one diagnosis code for a history of cancer or a secondary cancer before cohort entry. For acute outcomes, a patient was only required to have one diagnosis code to be included. A patient was considered to have an acute infection comorbidity if they had an infection code in the year prior to cohort entry. We required multiple diagnosis codes and supporting treatments or symptom codes for meningitis diagnoses in the DOD. Hospitalized infections were inpatient claims (DOD) or diagnosis codes with a hospitalization code within 1 week (CPRD). Suicidal behaviors were defined as having at least one code for suicidal ideations, suicide attempts, intentional self-harm or overdose.

Concomitant medications, recorded in the year prior to MS diagnosis date or matched date in the non-MS patients, included treatments for MS symptoms, as well as treatments for cardiovascular disease and other comorbidities. Many anticonvulsant therapies are indicated for both epilepsy and MS symptoms. We categorized anticonvulsant therapies as epilepsy treatments when the patient had a current or prior diagnosis of epilepsy. Otherwise, we categorized anticonvulsant therapies as treatments for MS symptoms.

### Statistical analysis

We described basic characteristics and study outcomes of the MS and non-MS patients at cohort entry (date of first recorded MS code/matched date). Proportions were compared using a chi-square test or, where cell sizes were less than 5, a Fisher’s exact test. Statistical analyses were carried out using SAS Release 9.3 (DOD study) and SAS Release 9.4 (CPRD study) (SAS Institute Inc., Cary NC, USA). Testing was not adjusted for multiplicity as this was a descriptive study of two independent large data sets exploring clinically relevant trends.

## Results

### DOD

We identified 8695 patients with MS and 86,934 matched non-MS patients in the DOD database. Most MS patients were female (71%), and a large portion were diagnosed before age 40 (45%) or between ages 50 and 59 (43%). See Table 2 for more details. Most cases were considered probable cases (N = 7172, 82.5%), i.e. they had many MS diagnoses (median = 45) and many disease-modifying treatment prescriptions (median = 30). Since we determined that in the DOD people with no disease-modifying treatments were not cases, there were few people who were considered only possible cases: 37 (0.4%) people had demyelinating disease codes with treatment and no MS codes, 125 (1.4%) people had five to nine MS diagnoses and five to nine MS disease-modifying treatments, and 1361 (15.7%) people had less than five MS diagnoses and/or less than five MS disease-modifying treatments. Possible cases had shorter records after the first MS diagnosis than probable cases (median 3.1 versus 7.5 years) which may account for a smaller number of diagnoses and prescriptions in this group. Among possible MS patients with records longer than 3 years, many had either few diagnoses of any kind in their records or had a few prescriptions for MS disease-modifying treatments and then stopped.

**Table 2:** Patient characteristics at time of first recorded MS diagnosis or matched date for non-MS patients, by database

	US—DOD		UK—CPRD	
	MS patients *N* = 8695 (%)	Non-MS patients *N* = 86.934 (%)	MS patients N = 6932 (%)	Non-MS patients *N* = 68.526 (%)
Age^a^				
< 40	3945 (45.4)	39,399 (45.3)	2742 (39.6)	27,356 (39.9)
40–59	3704 (42.6)	37,057 (42.6)	3320 (47.9)	32,816 (47.9)
60 +	1046 (12.0)	10,481 (12.1)	870 (12.5)	8354 (12.2)
Median (range)	41 (7–85)	41 (7–85)	43 (2–89)	43 (1–90)
Sex^a^				
Female	6205 (71.4)	62,037 (71.4)	4869 (70.2)	48,118 (70.2)
Male	2490 (28.6)	24,897 (28.6)	2063 (29.8)	20,408 (29.8)
Calendar year of cohort entry^a, b^				
2001–2005	1764 (20.3)	17,628 (20.3)	2407 (34.7)	23,871 (34.8)
2006–2010	3608 (41.5)	36,078 (41.5)	2437 (35.2)	24,098 (35.2)
2011–2017	3323 (38.2)	33,228 (38.2)	2088 (30.1)	20,557 (30.0)
Length of record before cohort entry				
1–2 years	2505 (28.2)	22,750 (26.2)	860 (12.4)	8039 (11.7)
3–5 years	2572 (29.6)	28,110 (32.3)	1088 (15.7)	11,074 (16.2)
6–9 years	2287 (26.3)	23,939 (27.5)	1420 (20.5)	13,759 (20.1)
10 + years	1331 (15.3)	12,135 (14.0)	3564 (51.4)	35,654 (52.0)
Median	5.0	5.1	10.3	10.4
Smoking				
Yes	2229 (25.6) *	12,836 (14.8)	1913 (27.6) *	15,341 (22.4)
Former	–	–	1628 (23.5)	14,008 (20.4)
No	1612 (18.5)	17,275 (19.9)	2852 (41.1)	32,327 (47.2)
Unknown	4854 (55.8)	56,823 (65.4)	539 (7.8)	6859 (10.0)
BMI				
Underweight < 18.5 kg/m^2^	51 (0.6)	396 (0.5)	174 (2.5)	1408 (2.1)
Normal weight 18.5–25 kg/m^2^	860 (9.9)	8410 (9.7)	2313 (33.4)	22,661 (33.1)
Overweight 25–30 kg/m^2^	945 (10.9)	9332 (10.7)	1861 (26.9)	18,315 (26.7)
Obese 30 + kg/m^2^	1813 (20.9)	11,431 (13.2)	1374 (19.8)	14,413 (21.0)
Unknown	5026 (57.8)	57,365 (66.0)	1210 (17.5)	11,729 (17.1)

*p < 0.0001

^a^Matching criteria

^b^The first year of US-DOD study was 2004. The last year of the UK-CPRD study was 2016

MS patients differed from the matched non-MS patients at cohort entry. MS patients had history of many more symptoms of MS including optic neuritis, disturbances of skin sensation, paresis, paralysis and muscle weakness, spasms/involuntary movements/lack of coordination, abnormality of gait, neuropathic pain and neuropathies, dizziness, etc. They were also more likely to have treated depression prior to the date of the first MS code or matched date in the non-MS patients (cohort entry date) compared to non-MS patients. See Table 3. MS patients were also slightly more likely to have had asthma or COPD, epilepsy, autoimmune disorders, hypertension, dyslipidemia, various cardiovascular diseases, and fracture. MS and non-MS patients were similar with respect to their history of other comorbidities. See Table 4. Finally, MS patients were more likely to have had many types of infections in the year prior to cohort entry compared to non-MS patients though the differences were small for many infection types. The greatest differences between MS and non-MS patients were found for urinary and kidney infections, and respiratory and throat infections. See Table 5. The use of medications that correspond to the treatment of MS symptoms and the comorbidities in the year before cohort entry differed between MS and non-MS patients in a predictable manner. MS patients received more drugs to treat movement disorders, pain, and fatigue. They also received more treatments for epilepsy, depression and other psychiatric conditions, and infections. See Table 6.

**Table 3:** MS symptoms at any time before first recorded MS diagnosis or matched date (cohort entry) among MS and non-MS patients, by database

Diagnosis codes^a^	US—DOD		UK—CPRD	
	MS patients *N* = 8695 (%)	Non-MS patients *N* = 86,934 (%)	MS patients *N* = 6932 (%)	Non-MS patients *N* = 68,526 (%)
Optic neuritis	876 (10.1)	117 (0.1)	842 (12.1)	48 (0.1)
Nerve disorder, unspecified	860 (9.9)	781 (0.8)	262 (3.8)	98 (0.1)
Paresis, plegia, paralysis, muscle weakness	1368 (15.7)	2646 (3.0)	987 (14.2)	668 (1.0)
Vestibular and labyrinthine disorders (ICD)/vertigo (Read codes)^a^	38 (0.4)	86 (0.1)	696 (10.0)	3235 (4.7)
Spasms, involuntary movements, lack of coordination, abnormality of gait (ICD)/spasticity or ataxia (Read codes) ^a^	1967 (22.6)	7586 (8.7)	629 (9.1)	1357 (2.0)
Treated depression	1875 (21.6)	13,459 (15.5)	1476 (21.3)	11,549 (16.9)
Neuritis and neuralgia (ICD also includes radiculitis)^a^	2214 (25.5)	8892 (10.2)	335 (4.8)	1075 (1.6)
Neuropathy	910 (10.5)	1778 (2.1)	200 (2.9)	193 (0.3)
Speech disorder	256 (2.9)	584 (0.7)	102 (1.5)	182 (0.3)
Disturbance of skin sensation (ICD)/paraesthesia, numbness or tingling (Read codes)^a^	4055 (46.6)	8125 (9.4)	2759 (39.8)	3580 (5.2)
Dizziness and giddiness	2225 (25.6)	9425 (10.8)	1161 (16.8)	5362 (7.8)
Malaise and fatigue	2657 (30.6)	17,090 (19.7)	1187 (17.1)	8951 (13.1)
Vision symptoms	1747 (20.1)	3565 (4.1)	598 (8.6)	690 (1.0)
Bladder symptoms	1861 (21.4)	13,581 (15.6)	971 (14.0)	6028 (8.8)
Bowel dysfunction ^b^	612 (7.0)	4237 (4.9)	640 (9.2)	5264 (7.7)
Pain, generalized	527 (6.1)	3001 (3.5)	1080 (15.6)	7629 (11.1)
Itch, unspecified	422 (4.9)	3521 (4.1)	186 (2.7)	1746 (2.6)
At least one of the symptoms above	7050 (81.1)	42,528 (48.9)	5627 (81.2)	31,646 (46.2)
At least two of the symptoms above	5647 (65.0)	23,993 (27.6)	3878 (55.9)	14,508 (21.2)

*p* < 0.001 for all MS versus non-MS comparisons within each database except for unspecified itch in CPRD GOLD

^a^Read and ICD codes are not all equivalent, so direct comparisons are not always possible

^b^Not including general constipation and diarrhea codes

**Table 4:** Important comorbidities at any time before first recorded MS diagnosis or matched date (cohort entry) among MS and non-MS patients, by database

Comorbidities	US—DOD		UK—CPRD	
	MS patients *N* = 8695 (%)	Non-MS patients *N* = 86,934 (%)	MS patients *N* = 6932 (%)	Non-MS patients *N* = 68,526 (%)
Treated depression	1875 (21.6)*	13,459 (15.5)	1476 (21.3)*	11,549 (16.9)
Other psychiatric diagnoses	853 (9.8)*	5850 (6.7)	51 (0.7)	486 (0.7)
Suicidal behaviors^a^	114 (1.3)*	815 (0.9)	354 (5.1)	3197 (4.7)
Asthma or COPD	450 (5.2)*	3901 (4.5)	1106 (16.0)*	10,081 (14.7)
Autoimmune disorders	23 (0.3)	153 (0.2)	389 (5.6)*	3250 (4.7)
Epilepsy	73 (0.8)*	376 (0.4)	157 (2.3)*	1065 (1.6)
Cancer	294 (3.4)	3014 (3.5)	164 (2.4)	1903 (2.8)
Breast cancer (among women)	78 (1.3)	989 (1.6)	41 (0.8)*	581 (1.2)
Prostate cancer (among men)	18 (0.7)	179 (0.7)	12 (0.6)	102 (0.5)
Liver disease	51 (0.6)	669 (0.8)	68 (1.0)	728 (1.1)
Treated diabetes	366 (4.2)	3933 (4.5)	112 (1.6)	1181 (1.7)
Treated hypertension	1699 (19.5)*	15,649 (18.1)	552 (8.0)	5756 (8.4)
Dyslipidemia	1012 (11.6)	10,059 (11.6)	387 (5.6)	3410 (5.0)
Myocardial infarction	17 (0.2)	309 (0.4)	45 (0.7)	513 (0.8)
Stroke	90 (1.0)*	256 (0.3)	56 (0.8)	469 (0.7)
Transient ischemic attack	120 (1.4)*	338 (0.4)	26 (0.4)	282 (0.4)
Venous thromboembolism	89 (1.0)	794 (0.9)	98 (1.4)	832 (1.2)
Peripheral vascular disease	21 (0.2)	199 (0.2)	31 (0.5)*	166 (0.2)
Bradycardia and heart block	9 (0.1)	88 (0.1)	19 (0.3)	221 (0.3)
Atrial fibrillation	36 (0.4)	391 (0.5)	47 (0.7)	427 (0.6)
Atrial flutter	6 (0.1)	53 (0.1)	NR	24 (0.0)
Other arrhythmias	84 (1.0)	656 (0.8)	107 (1.5)	999 (1.5)
Angina	36 (0.4)	492 (0.6)	90 (1.3)	942 (1.4)
Heart failure	23 (0.3)	405 (0.5)	26 (0.4)	234 (0.3)
Osteoporosis	63 (0.7)	647 (0.7)	69 (1.0)	594 (0.9)
Fracture	372 (4.3)*	3071 (3.5)	1485 (21.4)*	13,526 (19.7)
Alcohol abuse	63 (0.7)	519 (0.6)	176 (2.6)	1818 (2.7)

*NR* not reportable, number too small

*p < 0.01 between MS and non-MS, within database

^a^Includes suicide attempts, suicidal ideation, intentional self-harm and, in the CPRD, overdose

**Table 5:** Infections in the year before first recorded MS diagnosis or matched date (cohort entry) among MS and non-MS patients, by database

Infection site or type	US—DOD		UK—CPRD	
	MS patients *N* = 8695 (%)	Non-MS patients *N* = 86,934 (%)	MS patients *N* = 6932 (%)	Non-MS patients *N* = 68,526 (%)
Hospitalized infections	177 (2.0)*	998 (1.2)	131 (1.9)*	533 (0.8)
Urinary and kidney infections	874 (10.1)*	6099 (7.0)	315 (4.5)*	1977 (2.9)
Skin infections	430 (5.0)*	3155 (3.6)	253 (3.7)	2147 (3.1)
Respiratory and throat infections	2065 (23.8)*	17,838 (20.5)	857 (12.4)*	7699 (11.2)
Pneumonia and influenza	181 (2.1)*	1640 (1.9)	66 (1.0)	512 (0.8)
Eye and ear infections	584 (6.7)*	4598 (5.3)	338 (4.9)*	2210 (3.2)
Meningitis and encephalitis	0 (0)	1 (0.0)	NR	8 (0.0)
Intestinal infectious diseases	–	–	58 (0.8)	437 (0.6)
Viral infectious diseases	711 (8.2)*	5373 (6.2)	129 (1.9)	1025 (1.5)
Fungal infectious diseases	596 (6.9)*	4663 (5.4)	260 (3.8)	2171 (3.2)
Other infections	883 (10.2)*	6182 (7.1)	20 (0.3)	132 (0.2)

*NR* not reportable, number too small

*p < 0.01 between MS and non-MS, within database

**Table 6:** Description of medication use for treatment of MS symptoms or other comorbidities in the year before first recorded MS diagnosis or matched date (cohort entry) by MS and non-MS patients, by database

Medication type	US—DOD				UK—CPRD			
	MS patients *N* = 8695		Non-MS patients *N* = 86,934		MS patients *N* = 6932		Non-MS patients *N* = 68,526	
	Any use (%)	Median^a^	Any use (%)	Median^a^	Any use (%)	Median^a^	Any use (%)	Median^a^
Spasticity treatments	1406 (16.2)*	1	4829 (5.6)	1	753 (10.9)*	2	2241 (3.3)	1
Anticonvulsants with no epilepsy diagnoses	1688 (19.4)*	2	5280 (6.1)	3	690 (10.0) *	3	1012 (1.5)	5
Anticonvulsants with at least one epilepsy diagnosis	148 (1.7)*	5	494 (0.6)	7	84 (1.2)*	8	561 (0.8)	8
Anti-Parkinson’s treatments	19 (0.2)*	2	74 (0.1)	3	81 (1.2)*	3	240 (0.4)	8
Steroids	2215 (25.5)*	1	11,153 (12.8)	1	739 (10.7)*	1	2378 (3.5)	1
Neuropathic pain treatments	1012 (11.6)*	2	2487 (2.9)	3	685 (9.9)*	3	1119 (1.6)	6
Opioids	3337 (38.4)*	2	23,293 (26.8)	1	1674 (24.2)*	2	9025 (13.2)	2
NSAIDs	3956 (45.5)*	2	28,848 (33.2)	2	1567 (22.6)*	1	9996 (14.6)	1
Other analgesics	1819 (20.9)*	1	11,149 (12.8)	1	792 (11.4)*	3	4699 (6.9)	4
Fatigue treatments	147 (1.7)*	3	189 (0.2)	3	59 (0.9)*	1	9 (0.0)	7
Generalized itch treatments	290 (3.3)*	1	1951 (2.2)	1	21 (0.3)	2	184 (0.3)	1
Antidepressants	2467 (28.4)*	4	14,489 (16.7)	4	1856 (26.8)*	4	9254 (13.5)	5
Antipsychotics	265 (3.1)*	2	1364 (1.6)	4	641 (9.3)*	1	2215 (3.2)	1
Antihypertensives	2089 (24.0)*	4	16,607 (19.1)	5	1102 (15.9)*	6	9754 (14.2)	7
Statins	1216 (14.0)*	3.5	10,476 (12.1)	4	514 (7.4)*	6	4429 (6.5)	6
Anticoagulants	152 (1.8)*	1.5	1147 (1.3)	3	60 (0.9)	5	487 (0.7)	6
Antiplatelets	155 (1.8)*	3	766 (0.9)	4	108 (1.6)*	3	520 (0.8)	5
Other CVD treatments	78 (0.9)*	1	539 (0.6)	1	83 (1.2)	4	905 (1.3)	3
Insulin	104 (1.2)	6	1111 (1.3)	5	74 (1.1)	8	623 (0.9)	8
Other oral hypoglycemics	380 (4.4)	4	3948 (4.5)	5	135 (2.0)	8	1429 (2.1)	8
Immunosuppressants^b^	55 (0.6)	4	434 (0.5)	5	63 (0.9)	4	751 (1.1)	5
Asthma and COPD treatments	916 (10.5)*	1	7293 (8.4)	1	737 (10.6)*	3	6551 (9.6)	2
Proton pump inhibitors	1378 (15.9)*	3	10,496 (12.1)	3	924 (13.3)*	2	6542 (9.6)	4
Antibiotics	4051 (46.6)*	2	33,549 (35.1)	2	2427 (35.0)*	1	20,510 (29.9)	1
Sexual dysfunction treatments^c^	211 (8.5)*	2	1186 (4.8)	3	140 (6.8)*	3	481 (2.4)	3
OCs and HRTs^d^	1871 (30.2)*	3	14,848 (23.9)	3	1272 (26.1)*	2	11,731 (24.4)	2

*NSAIDs* Nonsteroidal anti-inflammatory drugs, *CVD* cardiovascular disease, *COPD* chronic obstructive pulmonary disease, *OC* oral contraceptives, *HRTs* hormone replacement therapies

*p < 0.01 between MS and non-MS, within database

^a^Median number of prescriptions among those with 1 or more prescriptions

^b^Immunosuppressants for autoimmune disorders

^c^Percentage among men

^d^Percentage among women

### CPRD Gold

We identified 6932 patients with MS and 68,526 matched non-MS patients in CPRD GOLD. The age at onset and sex distribution of MS cases are similar in the CPRD and the DOD data (see above). See Table 2 for details. After receiving questionnaires for a sample of all MS patients to validate the diagnoses, we calculated a positive predictive value (PPV) of 92% overall. The PPV was 100% for probable MS patients (53.5% of all MS patients), 84% among possible MS cases and 56% among unlikely MS patients. As in the DOD data, MS patients differed from the matched non-MS patients at the date of the first MS code (cohort entry), or the matched date in the non-MS patients, in many ways. MS patients had history of many more symptoms of MS including optic neuritis, nerve disorders, ataxia, and paresthesias, and more neurology referrals and visits. See Table 3. They were also more likely to have treated depression prior to the cohort entry date compared to non-MS patients. Differences in the prevalence of other comorbidities, including acute infections, were also similar to the DOD findings. See Tables 4 and 5. The greatest differences in infection rates were found for urinary and kidney infections, and eye and ear infections. The use of medications that correspond to the treatment of MS symptoms and the comorbidities in the year before cohort entry were more prevalent in the MS patients, and were similarly different in the CPRD GOLD and the DOD databases. See Table 6.

## Discussion

This study used data from the CPRD GOLD and DOD, both valuable databases for the conduct of disease epidemiology studies, to characterize MS patients and matched patients who did not have MS, with respect to symptoms, comorbidities, and treatments at the time of the MS diagnosis (or the matching date in the non-MS patients). The results of this study are consistent with what is known about early MS symptoms and their treatments, and MS comorbid conditions. In addition, new associations were observed that will add valuable clinical insights to support earlier disease diagnosis and treatment. For example, fracture was more common at cohort entry in MS compared to non-MS patients as was asthma/COPD. Breast cancer was less common in MS compared to non-MS patients and cardiovascular disease was similar in MS and non-MS patients at the time of MS diagnosis. Future analyses of these patients’ experience after MS diagnosis will provide valuable insights into disease and treatment patterns in relation to risk of chronic diseases and mortality. Note that the results of this study address comorbidities of MS at the time of the first recorded MS diagnosis. We did not attempt to identify the date of MS onset for this study so all results should be interpreted accordingly. This study adds to the knowledge of signs, symptoms and concomitant disease present when the MS diagnosis has been made and is not a study of causes of MS.

While the findings of this study are broadly similar to earlier findings, there are some apparent differences between US and UK patients in both the MS and non-MS cohorts. For example, MS patients tend to be diagnosed at a younger age in the US compared to the UK. In the US, the most common comorbidities present in MS patients at cohort entry were treated depression, treated hypertension, fracture, dyslipidemia and ‘other psychiatric disorders’. The most common comorbidities in the UK MS population, at cohort entry, were fracture and treated depression, followed by asthma/COPD, treated hypertension and dyslipidemia. It should be noted that asthma was more prevalent in the UK while hypertension was more prevalent in the US in both MS and non-MS patients. These differences are likely to reflect a combination of differences in the health care systems, the source of data (Claims versus EMR), as well as differences in the US and UK populations. It is also possible that the longer look back in the CPRD (data go back as far as 1988 for some patients) could explain the higher prevalence of certain chronic conditions in the CPRD. Other conditions that were less prevalent overall but more common in MS patients compared to non-MS patients include epilepsy and venous thromboembolism (in the DOD) or peripheral vascular disease (in the UK). Certain terms including vestibular and labyrinthine disorders/vertigo, neuritis/neuralgia/radiculitis, and disturbance of skin sensation/paraesthesia, numbness or tingling are grouped differently in the DOD compared with the CPRD (ICD versus Read), demonstrating that, for some conditions, direct comparison of frequencies between DOD and CPRD patients is difficult to interpret.

Our results generally agree with the few prior reports that evaluated comorbidities at time of MS diagnosis. In the current study, in both databases, the prevalence of depression in MS patients (~ 21%) was higher than in non-MS patients, as was found in a Canadian study that reported 19.1% prevalence of depression at MS diagnosis [16]. The prevalence of other psychiatric diagnoses at MS diagnosis was also higher compared to non-MS patients in Canadian [16] and French MS patients [17]. Finally, a Danish study reported an odds ratio of 1.4 (95% confidence interval (CI) 1.05–1.88) for diagnosis of depression or anxiety during the 2 years prior to MS diagnosis [18].

In both the US and UK databases, the prevalence of treated diabetes and dyslipidemia at MS diagnosis were similar for MS and non-MS patients. In the CPRD only, the prevalence of treated hypertension was also similar in MS and non-MS patients. Treated hypertension was marginally higher in MS patients in the DOD cohort compared to non-MS patients. In the Canadian study, the prevalences of hypertension, diabetes and dyslipidemia at MS diagnosis were 15.2%, 5.69%, and 6.89%, respectively, and both hypertension and diabetes (but not dyslipidemia) had higher prevalence in MS compared to non-MS patients [16]. Among French patients aged 15–45, the combined prevalence of Type I and Type II diabetes was substantially higher in MS patients (18.5%) than non-MS patients (8.6%) [17]. In our study, the age range was up to age 90 and we reported on type II diabetes only; therefore, the results of these studies are not comparable.

In both cohorts in our study, the prevalence of epilepsy at MS diagnosis was approximately twice as high as non-MS patients, as was seen in the Canadian study (MS: 1.93% vs. non-MS: 0.89%) [16]. Similarly, in both the CPRD and DOD cohorts, the prevalence of “asthma or COPD” was higher for MS than non-MS patients (5.2% vs 4.5% and 16.0% vs 14.7%), consistent with the finding of the Canadian study, which reported a higher prevalence of chronic lung disease at MS diagnosis versus non-MS patients (12.1% vs. 9.14%) [16].

Other diagnoses common in MS patients in both the US and the UK data included multiple types of infections such as urinary and kidney, respiratory and throat, and eye and ear. All were significantly more common amongst the MS compared to the non-MS patients in the year prior to the MS diagnosis. Although the prevalence and incidence of infections are higher among MS patients after diagnosis [19], our study adds important new evidence of heightened infection risk prior to MS diagnosis. One of the findings, an increased risk of urinary tract infections, could be due to MS-related reduction of muscular control, but there may be alternate explanations. Although many studies have reported associations of bacterial and viral infections with subsequent MS, in a meta-analysis, only Epstein–Barr virus (and seropositivity to EBV nuclear antigen (anti-EBNA IgG)) and infectious mononucleosis have consistent positive associations with MS [20].

The presence of many MS symptoms at the time of MS diagnosis was predictably common among MS patients in both the US and UK data resources. Results were consistent with a recent meta-analysis that reported the following prevalences in patients with existing MS: neuropathic pain 28.5%, painful spasms 15.0%, and trigeminal neuralgia 3.8% [21]. Neurologic conditions, such as optic neuritis, paresthesia, and dizziness, were similarly common prior to the diagnosis of MS, as expected, in both the US and UK. Optic neuritis is often the presenting symptom of MS, and longitudinal studies show that 34–75% of patients presenting with optic neuritis in the UK and US develop MS [22, 23] and a relative risk > 30 for MS for Chinese individuals with optic neuritis followed for 9 years [24]. The prevalences of other symptoms such as treated depression and malaise or fatigue were also similar in the two databases. Likewise, the treatments for these symptoms were common among MS patients in both the DOD and CPRD. Use of drugs to treat MS symptoms was notable in that a higher proportion of MS patients in the DOD compared to the CPRD received drugs for MS symptoms including spasticity, convulsions, and pain. Use of pain medications was also more common in non-MS patients in the DOD versus the CPRD. Recognition of patterns of symptoms and treatments that manifest at MS onset but before first diagnosis could lead to earlier diagnosis and treatment for MS patients and potentially improved long-term prognosis. Use of statins, steroids, and antibiotics was also higher in the DOD for both MS and non-MS patients which may reflect a general tendency toward higher prescribing in the US compared to the UK.

The DOD and CPRD databases each have their own strengths and limitations. We used different criteria to identify MS patients in the DOD and CPRD databases. In the US DOD database, patients with confirmed MS are treated with MS-specific therapies. Hence, the inclusion of MS treatment records as a requirement in the case definition. In the UK, most MS treatments are not recorded on GP computers because they are not outpatient prescriptions but rather infusions given in specialty clinics or are prescribed by specialists. In addition, not all people with MS receive MS-specific treatment in the UK. Furthermore, while the nature of claims data leads to repeated entries for chronic diseases such as MS in the DOD database, the EMR character of the CPRD results in as few as one or two codes for chronic conditions over a span of many years. Thus, in the CPRD, the MS case definition did not include MS treatment, nor multiple MS records as required conditions. On the other hand, the CPRD does contain supplementary codes for symptoms and services related to MS which were used to identify likely cases. These differences required different validation processes and inclusion criteria to identify “true” MS cases in the two databases. Despite these differences in the database-specific case criteria, we were able to identify populations of MS patients that we validated through questionnaires or electronic record review in both data sources. The similarity in results between the two data resources provides confidence in the case selection process. Despite this, it is possible that some patients who did not have MS were included in the study. However, it is unlikely that there were many such cases or that their inclusion had much influence on the study results. The CPRD has been used for many studies of MS in the past where extensive validation was done [8–15]. These studies demonstrated the high predictive value of the MS case definition applied in this study. There has been one prior study of MS in the DOD database [4].

Information on patient characteristics such as smoking and BMI was available for most patients in the CPRD (> 80%) but not in the DOD where data were only available for around 35%. Thus, evaluation of these associations to MS will be restricted to the CPRD data.

Information on treatments for MS and concomitant medications is available in the DOD data and includes outpatient prescriptions as well as infusions and injections. Concomitant medications are also captured in the CPRD and treatments for MS symptoms were noted for many patients. However, a limitation of the CPRD is the absence of treatments administered outside the GP office. Thus, most MS treatments were not captured in the CPRD portion of the study. This limitation does not impact the results provided here but will have implications for further analyses of MS outcomes according to MS treatments.

The DOD database in the US and the CPRD in the UK constitute valuable resources for the study of MS patients, their symptoms, comorbidities, and medication use at diagnosis and after. Long follow-up will enable us to describe the prognosis of MS patients compared to patients without MS over time.
